# Micro-Actuated Tunable Hierarchical Silver Nanostructures to Measure Tensile Force for Biomedical Wearable Sensing Applications

**DOI:** 10.3390/mi12050476

**Published:** 2021-04-22

**Authors:** Yong Ho Kwon, Jayer Fernandes, Jae-Jun Kim, Jiangang Chen, Hongrui Jiang

**Affiliations:** 1 Department of Electrical and Computer Engineering, University of Wisconsin-Madison, Madison, WI 53706, USA; ykwon9@wisc.edu (Y.H.K.); jfernandes@wisc.edu (J.F.); jkim724@wisc.edu (J.-J.K.); jiangang.chen@wisc.edu (J.C.); 2 Department of Materials Science and Engineering, University of Wisconsin-Madison, Madison, WI 53706, USA; 3 Department of Ophthalmology and Visual Sciences, University of Wisconsin-Madison, Madison, WI 53706, USA; 4 Department of Biomedical Engineering, University of Wisconsin-Madison, Madison, WI 53706, USA; 5 McPherson Eye Research Institute, University of Wisconsin-Madison, Madison, WI 53706, USA

**Keywords:** nanoisland, surface plasmon, flexible, tunable, photonic sensor, visible spectrum

## Abstract

Commercially available biomedical wearable sensors to measure tensile force/strain still struggle with miniaturization in terms of weight, size, and conformability. Flexible and epidermal electronic devices have been utilized in these applications to overcome these issues. However, current sensors still require a power supply and some form of powered data transfer, which present challenges to miniaturization and to applications. Here, we report on the development of flexible, passive (thus zero power consumption), and biocompatible nanostructured photonic devices that can measure tensile strain in real time by providing an optical readout instead of an electronic readout. Hierarchical silver (Ag) nanostructures in various thicknesses of 20–60 nm were fabricated and embedded on a stretchable substrate using e-beam lithography and a low-temperature dewetting process. The hierarchical Ag nanostructures offer more design flexibility through a two-level design approach. A tensional force applied in one lateral (*x-* or *y-*) direction of the stretchable substrate causes a Poisson contraction in the other, and as a result, a shift in the reflected light of the nanostructures. A clear blue shift of more than 100 nm in peak reflectance in the visible spectrum was observed in the reflected color, making the devices applicable in a variety of biomedical photonic sensing applications.

## 1. Introduction

Flexible electronics is an emerging field that has huge potential in biomedical wearable sensing applications due to its wearability and implantation capabilities [1,2,3,4,5,6,7,8,9,10]. A number of disorders, such as glaucoma, repetitive strain injury (RSI), cardiomyopathy, axonal injury, etc., which suffer from a limited understanding of their pathophysiology due to the lack of sensors for measuring required data, could benefit tremendously from flexible sensing techniques. The potential causes of these disorders are closely related to continuously applied strains in joints, ligaments, and muscles that hold the internal organs [11,12,13,14,15,16,17]; hence, monitoring the changes of tension forces in real time via flexible sensors could provide much needed data on these strains. Nevertheless, current flexible sensors are not suitable for directly and accurately monitoring such tension forces. Their relatively large sizes make it challenging to attach them to the structural surfaces of the joints, ligaments, and muscles. In addition, the requirement of electronic accessories such as power supplies and data transfer modules for wireless communication further increases the overall weight of the flexible sensors, hindering many of their potential wearable applications. In general, for electronics-based flexible sensors in wearable sensing applications, in order to provide electronic data, they often require more complex designs, induce higher costs, sacrifice flexibility, and increase overall sensor weight and size. For example, achieving epidermal characteristics with electronics-based hardware is an actively researched area, with major challenges in reducing the size and weight of the devices and sensors. On the other hand, providing a passive means of data readout, such as optical methods instead of electronic methods, could be a promising step towards solving these issues by reducing the complexity of the design, lowering costs, improving flexibility, and reducing the size and weight of the sensor.

In this study, we developed a tunable photonic nanostructure embedded in a membrane that can, when laterally actuated (*x-* or *y*-direction), detect and measure the tensile strain and passively provide data about such strain. ANSYS simulations were conducted to understand the behavior of the tunable membrane during the micro-actuation. Dewetted silver (Ag) nanoislands were embedded inside the cavity of the membrane, and the plasmon resonance effect was utilized to take advantage of the blue shift of the reflected color as a lateral strain was applied to the membrane. Each cavity in the membrane was 200 nm in width, 200 nm in length, and 100 nm in height. Different thicknesses of dewetted Ag nanoislands were deposited and embedded, from 20 nm to 60 nm, to realize the optimized dimensions to achieve the highest peak reflectance of the sensor. As a tensile strain was applied to the sensor, the reflected color in the visible spectrum from the dewetted Ag nanoislands changed corresponding to the tensile strain applied to the sensor membrane; this color change could be read out optically. Finite difference time domain (FDTD) simulations were conducted to understand the shift of the reflected color due to the change in the period of the cavities and nanoislands.

Nanostructured photonic devices have been developed for and utilized in various applications, including displays [18], lighting [19,20], solar cells [21,22], mechanical sensors [23,24], and biosensors [25]. The optical properties of photonic nanostructures depend heavily on their geometric parameters; consequently, they can be manipulated by changing the nanostructures’ designs [26,27,28]. Although photonic nanostructures can be optimized for and utilized in specific applications, on the downside, the predefined physical structures have a limited tuning capability and working bandwidth that could restrict potential applications [29,30]. To overcome these limitations, tuning techniques for changing the geometric parameters of photonic nanostructures have been studied. Fabricating photonic nanostructures on stretchable substrates is the main method to tune their optical properties by changing their geometric parameters through the use of different actuation mechanisms [31,32,33].

**Figure 1:**
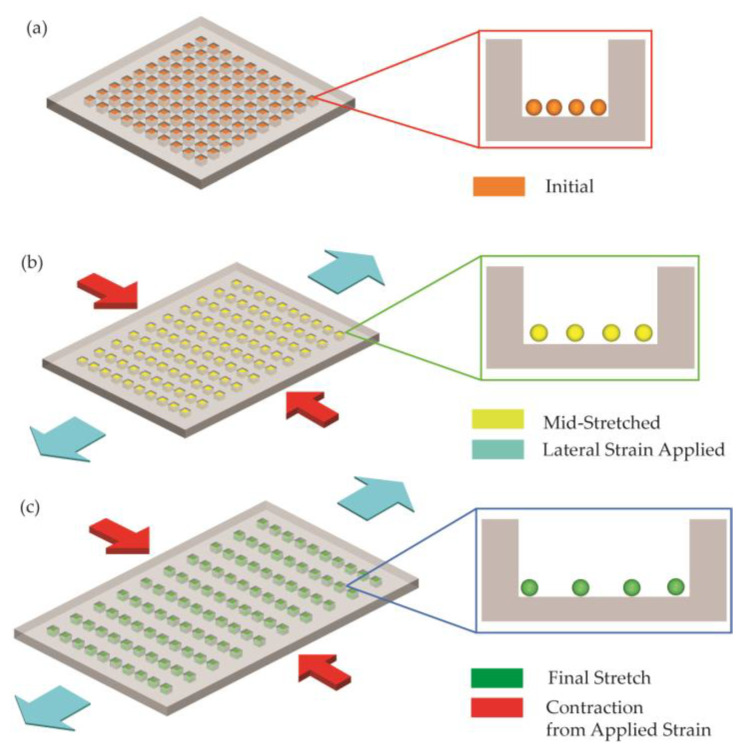
Illustration of the change in the reflected color of hierarchical dewetted Ag nanostructures (Ag nanoislands formed in nanowells) on a stretchable substrate before (**a**) and after (**b**,**c**) being stretched. The schematics indicate the Poisson contraction of the membrane in the direction orthogonal to the applied strain.

In order to apply nanostructured photonic devices to wearable sensing applications, the limitation in the tuning capability and working bandwidth must be resolved. Otherwise, the sensor will not be able to detect a wide range of tensile force/strain applied to the sensing area. Therefore, this work used micro-actuation of the flexible sensor membrane to achieve tunability to cover a wide range in the visible spectrum. Figure 1 shows the schematic of the hierarchical Ag nanostructures fabricated on a stretchable substrate. As tension/compression is applied, it changes the dimensions of the hierarchical Ag nanostructures, resulting in a shift in color of the reflected light from the nanostructures. Furthermore, if a tension force/strain is applied in the one direction (e.g., *z*-direction in Figure 1), there is a compression in the other two orthogonal directions (e.g., *x*-direction in Figure 1) due to Poisson contraction. Therefore, combining hierarchical Ag nanostructures with a thin-film elastomeric material could lead to a highly sensitive photonic sensing mechanism, due to the dependence of the visible color of the reflected light on the applied strain on the membrane. Utilizing a polymer-based material that possesses flexible, epidermal, and biocompatible characteristics as the supporting substrate makes this photonic sensing scheme especially suited to biosensing applications.

## 2. Materials and Methods

### 2.1. Principle and Structure

**Figure 2:**
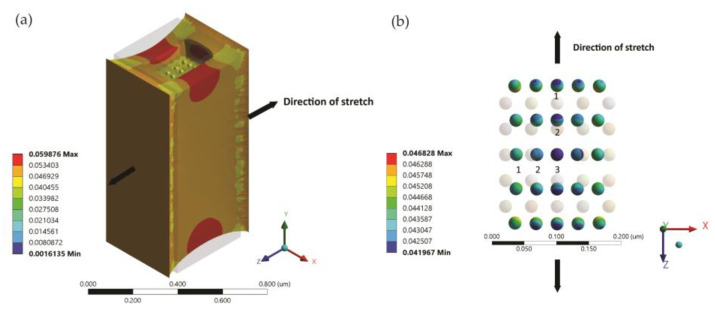
Illustration of the ANSYS simulation. (**a**) Schematic of the simulated device structure, indicating the direction of the applied uniaxial strain. The undeformed model is also shown. A displacement boundary condition of 50 nm was applied in this case. (**b**) Displacement of the nanoisland array under an applied displacement of 50 nm, showing the movement in the *x* and *z*directions in relation to the original positions.

Finite element ANSYS simulations using the ANSYS Mechanical/Static Structural module were conducted to better understand the behavior of the nanostructure under unilateral strains and to validate our hypothesis behind the observation of the blue shift behavior of the reflected spectrum under strain. As shown in Figure 2, we modeled a single unit cell of two-dimensional nanograting. The unit cell had a face area of 400 nm × 400 nm, with a thickness of 1 μm. A well with a face area of 200 nm × 200 nm and a depth of 100 nm was created in the PDMS structure. The nanoisland structure was approximated by using a periodic two-dimensional array of Ag hemispheres, with a diameter of 20 nm and a spacing of 20 nm in both *x-* and *z*-directions (thus a period of 40 nm). The material used to define the material properties of the structure was PDMS, with a Young’s modulus of 5 × 10^5^ Pa and a Poisson’s ratio of 0.4 [34]. A uniaxial displacement boundary condition was applied to the unit cell in both the positive and negative *z*-directions and varied from 0 μm to 1 μm. The movement of the nanoislands in the direction of the stretch (*z-*direction) and the direction transverse to the stretch (*x*-direction) was tracked by locating the position of their centroids (marked as centroid 1, centroid 2, and centroid 3). The resulting change in the spacing between the nanoislands in the *x-* and *z*-directions was plotted as a function of the uniaxial strain.

**Figure 3:**
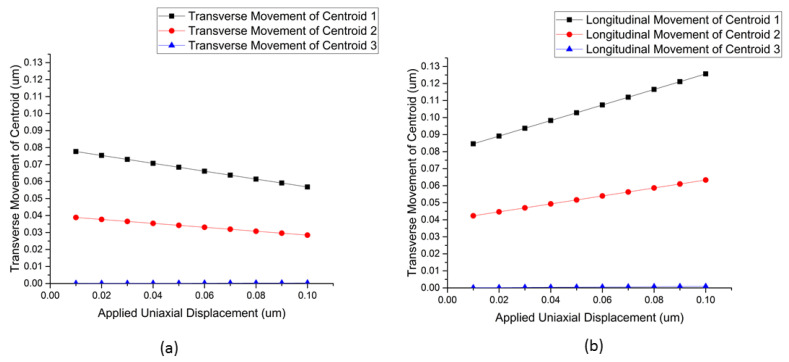
(**a**) Displacement of the centroids of nanoislands 1, 2, and 3 in the transverse direction of a stretch, indicating a reduction in the spacing under an applied strain. (**b**) Displacement of the centroids of nanoislands 1, 2, and 3 in the direction of the strain, indicating an increase in the spacing under an applied strain.

The results in Figure 3 show that as the displacement was increased in the *z*-direction, the device experienced a Poisson’s contraction in the transverse (*x-*) direction. This had the effect of bringing the nanoislands closer together in the transverse (*x-*) direction, while significantly increasing their spacing in the direction of the stretch (*z*-direction). The plasmonic effect has an inverse distance relationship, the effect of which is dominated by the size of the gaps in relation to the wavelength of the incoming light [35].

The optical response of conventional plasmonic structures has a strong dependence on the size and shape of the metal nanostructure, the metal used, and the refractive index of the surrounding medium. Light incident on these nanoparticles results in oscillations of their electrons, resulting in resonances known as localized surface plasmons [36]. Coupled nanoparticles at close distances show intense optical fields in the interparticle gaps. As a result, it is possible to achieve spectral tuning by varying the interparticle distance using appropriate actuation mechanisms [37]. In order to maximize the plasmonic effect, a contraction movement is desired in order to bring the particles closer. However, flexible polymer-based sensors are limited in their physical ability to contract without warping. Therefore, our design principle is focused on stretching in one direction, so that an orthogonal (transverse) direction will experience a contraction due to the Poisson effect.

**Figure 4:**
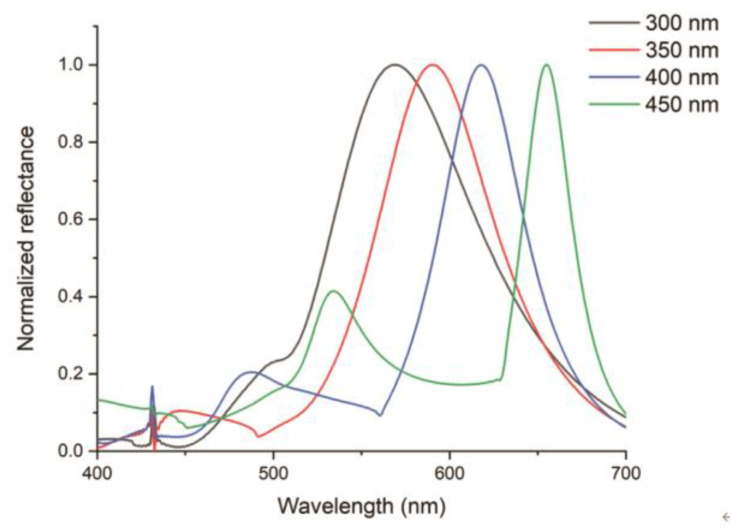
FDTD simulation results of the change of peak reflectance of Ag nanoislands with respect to the change of their spatial period (300 nm, 350 nm, 400 nm, and 450 nm, respectively).

The sensor structure comprised an array of dewetted Ag 2D nanoislands embedded in a flexible and biocompatible 100 μm thick polydimethylsiloxane (PDMS) membrane. Ag provides high reflectance in the visible wavelength range between 400 nm and 700 nm. The relatively weak adhesion force of Ag makes it easier to selectively remove from the surface after deposition. The structural color in the visible range stemming from the surface plasmon resonance of the metallic 2D nanoislands provided information about the change in the tensile force. A 2D nanoisland structure was chosen in order to accommodate the radial expansion of the sensor membrane along lateral directions in response to a tensile force change, allowing for the shift in the period of the nanoislands along both directions, resulting in vivid color patterns [38,39,40,41,42,43]. As shown in Figure 4, as the period of the Ag nanoislands decreased, the peak reflectance moved towards the blue portion of the visible spectrum. When a lateral *z*-direction tensile force was applied, the *z*-direction stretched while the *x*-direction contracted. In this case, the plasmonic coupling in the contracted *x*-direction was the more dominant effect. Based on the simulation results, we could expect a clear blue shift of the reflected color from the PDMS membrane when a lateral tensile force is applied.

### 2.2. Fabrication

We fabricated hierarchical Ag nanostructures on a stretchable substrate utilizing electron-beam (e-beam) lithography and solid-state thermal dewetting. Ag is a suitable material to fabricate nanoislands for visible wavelength applications. The hierarchical Ag nanostructures were designed at two different levels: (1) nanostructures defined by e-beam lithography were used to fabricate nanowells, and (2) Ag nanoislands were formed in the nanowells by solid-state thermal dewetting. This two-level design approach could increase the number of design parameters and thus offer more freedom and flexibility in design. We also demonstrated the use of a low-temperature Ag dewetting process (80 °C). Conventional nanoisland formation takes place at high temperatures, mostly above 110 °C and up to 700 °C [44,45]. The benefits of fabrication at lower temperatures include reduced polymer expansion, less distortion, and fewer defects, which in turn improve the compatibility between the polymer-based substrate material and the metal nanoislands.

**Figure 5:**
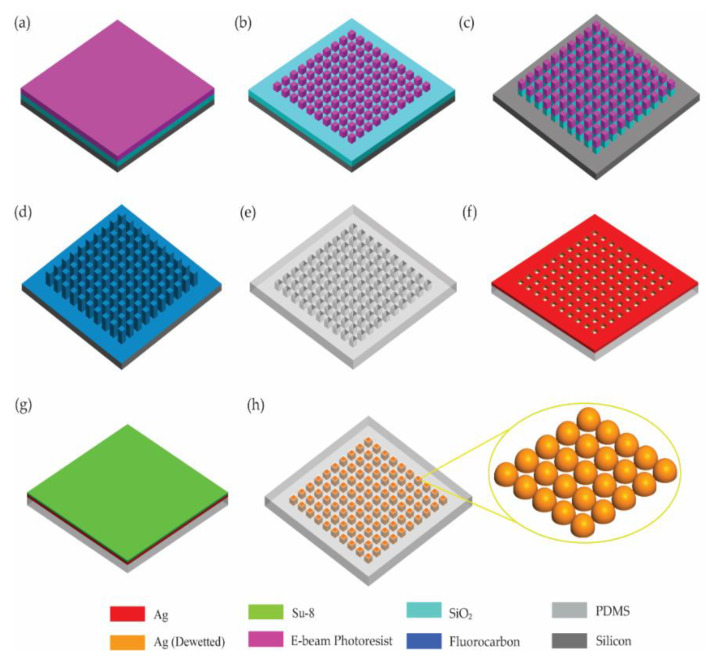
Illustration of the fabrication process of Ag nanoislands in nanowells embedded in a PDMS membrane. (**a**) SiO_2_ deposition on a Si wafer, followed by spin coating of an e-beam photoresist. (**b**) E-beam lithography. (**c**) RIE to generate a nanowell template. (**d**) Fluorocarbon coating to render the template surface hydrophobic. (**e**) Fabrication of a PDMS mold from the template. (**f**) Ag deposition into the PDMS nanowells. (**g**) Dewetting with SU-8 spin coated on a glass slide. (**h**) Removal of the SU-8.

The fabrication of the Ag nanoislands took place on a silicon (Si) substrate and was then transfer-printed to a flexible polymer-based (PDMS) membrane. First, a 200 nm thick layer of silicon dioxide (SiO_2_) was deposited on bare Si via plasma-enhanced chemical vapor deposition (PECVD). A mixture of 900 SCCM of N_2_O and 400 SCCM of 2% silane gas was used under a 600 mT pressure at 350 °C to deposit SiO_2_ at 450 Å/min. As the surface of SiO_2_ is extremely hydrophilic, HMDS (hexamethyldisilazane) processing took place on the deposited SiO_2_ to convert its surface to hydrophobic. Once the substrate was prepared as shown in Figure 5a, a negative e-beam photoresist (ma-N 2401, Micro Resist Technology) was spin coated on the deposited SiO_2_ with a thickness of 100 nm. An e-beam lithography step was performed next to create a periodic template mask of square nanopillars as shown in Figure 5b.

After the photoresist was developed, each photoresist nanopillar was 200 nm in width, 200 nm in length, and 100 nm in height. The photoresist nanopillars were used as masking layers to create SiO_2_ nanopillars. Reactive ion etching (RIE) was then performed to anisotropically etch the SiO_2_ and create a periodic array of pillars with physical dimensions of 200 nm width, 200 nm length, and 200 nm height with a 50% duty cycle as illustrated in Figure 5c. Mixed gas of argon (Ar) and trifluoromethane (CHF_3_) was applied under 5 mT pressure and 200 W to etch SiO_2_ at a rate of 200 Å/min. High-power oxygen plasma treatment was performed to remove photoresist layers and residue from dry etching. High-power ultrasonication was performed in acetone to clean any leftover residue. The plasma-exposed Si substrate, photoresist, and deposited SiO_2_ were hydrophilic; therefore, a fluorocarbon coating was performed using an STS deep reactive ion Si etcher (Figure 5d) to achieve hydrophobic characteristics. A polydimethylsiloxane (PDMS) mold was subsequently created based on this template, as shown in Figure 5e, resulting in a periodic array of nanowells. Depending on the target application, the thickness of the PDMS can be varied. In this fabrication process, the PDMS thickness was designed to be less than 1 mm in order to facilitate measurement.

Once the PDMS mold was created, Ag with a thickness of a few tens of nanometers was deposited using metal evaporation directly on top of the mold as shown in Figure 5f. At this point, Ag was deposited in the cavity of the nanopillars and on the surface of the PDMS mold. A microscope cover glass slide with spin coated SU-8 (2000.5) was aligned on top of the PDMS mold and heated to 80 °C to achieve the dewetting of Ag and the final formation of the Ag nanoislands, as well as the removal of unwanted surface layers on the PDMS (Figure 5g). The area of each array was 100 × 100 μm^2^ and the nanowells were densely packed in each array. Removing the SU-8 film cleaned the residues on the surface of the mold and completed the Ag nanoislands embedded in the flexible PDMS membrane for photonic sensing application, as illustrated in Figure 5h.

### 2.3. Experimental Setup

The thin film with dewetted Ag nanoisland structures that we developed can be applied to biomedical photonic sensing applications, in which the tunability of the nanoisland structure is key. We prepared an experimental setup to test the tunable characteristics of the Ag nanoisland structure. The dewetted Ag nanoislands were embedded in the thin PDMS membrane, and each side of the membrane was tightly clamped under a microscope. The edges were manually stretched in one direction as shown in Figure 3. The sensor was illuminated under normal incidence using a microscope lamp (LV-HL50 W Nikon Halogen Bulb). A 20× objective lens was set up to collect the reflected light from the stretched membrane.

**Figure 6:**
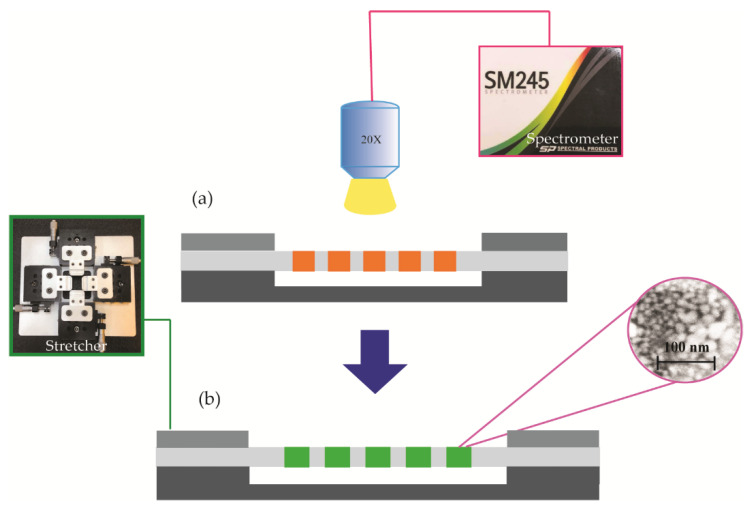
Schematic of the characterization setup for the thin film (a flexible PDMS membrane) with Ag nanoislands embedded, (**a**) before and (**b**) after stretching the PDMS film. Inset, SEM image of the dewetted Ag nanoislands.

Scanning electron microscope (SEM) images were taken to verify the dewetting of the Ag nanoislands. For the purpose of this verification, the fabrication process as described in Figure 5 was slightly modified. After the Ag was deposited onto the PDMS as shown in Figure 5g, a thicker layer (on the order of 1 µm) of SU-8 (2010) was used instead of SU-8 (2000.5) of nanometer-scale thickness. Furthermore, the PDMS membrane was adjusted to be 100 μm in thickness to allow SU-8 (2010) to flow inside the cavity of the membrane. Using SU-8 (2010) and lowering the PDMS membrane thickness allowed us to remove the dewetted Ag nanoislands from inside the nanowells of the membrane for the purpose of SEM imaging. The inset in Figure 6 shows the SEM image of the dewetted Ag islands that were transferred to the SU-8 (2010) film.

We measured the reflectance spectra of the nanostructures using a custom-built microscope–spectrometer combination. To set up the microscope–spectrometer, we added a Nikon Y-IDP double port that had a beam splitter (55(front):45(rear)) to a microscope (Nikon LV 100) and coupled a CCD spectrometer (SM 245, Spectral Products Inc., wavelength range: 380–760 nm) to the rear port of the Y-IDP double port using a multimode optical fiber. A CMOS camera was coupled to the front port of the Y-IDP double port. Both the CMOS camera and the optical fiber were placed at the image plane of the optical system.

## 3. Result and Discussions

### 3.1. Microscope Image

**Figure 7:**
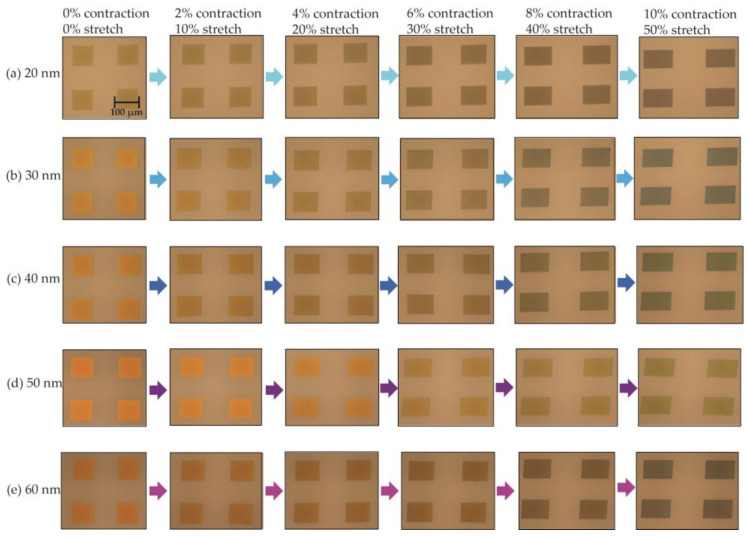
Optical microscopic images of the visible color change due to applied lateral strain on the PDMS membrane involving up to 50% stretching in the lateral direction, and 10% contraction in the vertical direction, with hierarchical Ag structures of different thicknesses of (**a**) 20 nm, (**b**) 30 nm, (**c**) 40 nm, (**d**) 50 nm, and (**e**) 60 nm.

We first tested the PDMS membrane with embedded Ag nanoislands under the custom-built microscope (Figure 7). Each square array in the membrane in Figure 7 was 100 × 100 μm^2^. Inside each square array, 200 nm by 200 nm dewetted nanoislands were densely packed with a 50% duty cycle. A variety of samples with different thicknesses of deposited Ag were prepared and dewetted to form nanoislands, and lateral tensile forces were applied to the membrane to characterize their optical behavior. After a lateral tensile force was applied to the dewetted Ag nanoislands embedded in the thin PDMS membrane, the reflected light from the nanoisland structure showed a clear blue shift as shown in Figure 7. The images were taken under the microscope after incremental stretches of 10% and contractions of 2% after a lateral tensile force was applied. The experiments were repeated by applying the same strain in both lateral directions separately, and a similar blue shift phenomenon was observed. The 20 nm deposited Ag (Figure 7a) was too thin, and its particles were too scattered to visibly detect a change of plasmonic effect. Deposited Ag with thicknesses between 30 and 60 nm showed a much clearer blue shift from orange–yellow to green–blue in the visible spectrum.

### 3.2. Spectroscopy Results

**Figure 8:**
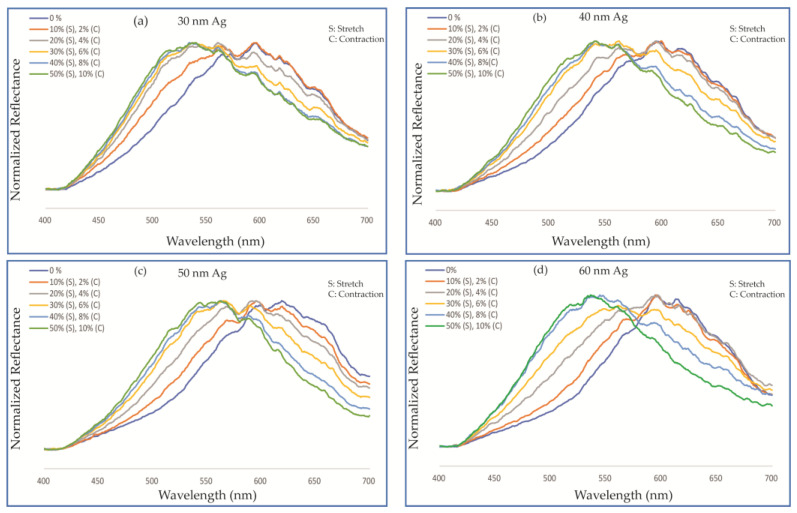
Normalized reflectance graph measured by a spectrometer for different thicknesses of deposited Ag: (**a**) 30 nm, (**b**) 40 nm, (**c**) 50 nm, and (**d**) 60 nm. The PDMS membrane underwent a strain from 0 to 50% in the direction being stretched and, as a result, a contraction from 0 to 10% in the other lateral direction.

We performed further spectroscopic characterization of our PDMS thin film with embedded hierarchical Ag nanoislands, utilizing our custom-built microscope–spectrometer. The spectrometer collected the reflected light from a small region (~20 μm) of the membrane, because the fiber core acts as a confocal pinhole. The spectrometer data in terms of intensity and normalized reflectance are plotted in Figure 8. The results in Figure 8 show a clear blue shift for deposited, dewetted Ag with thicknesses of 30, 40, 50, and 60 nm, which corroborates the microscopy results. As a tensile force was applied to stretch the PDMS membrane up to 50% in the lateral direction and cause a contraction of up to 10% in the vertical direction, a blue shift was confirmed in both the intensity and normalized reflectance graph. As the thickness of the deposited Ag increased, it showed a larger shift in the spectrometer plots. The thicker initially deposited Ag created larger individual dewetted Ag nanoislands and decreased the distance between nanoislands, which ultimately determined the initial plasmon resonance wavelength closer to the red color [32]. The most significant change occurred between 50 and 60 nm thickness, where about 100 nm of blue shift was observed.

The results can be qualitatively explained as follows. Stretching the PDMS membrane uniaxially resulted in an increase in the distance between the dewetted Ag particles in the direction of the stretch. This in turn reduced the coupling between them, and their contribution to the resonance effect diminished significantly. On the other hand, since PDMS has a Poission ratio of approximately 0.49, the uniaxial stretch in one direction was accompanied by a compression of the membrane in the transverse direction. This compression resulted in the dewetted Ag nanoparticles coming closer together in the transverse direction, resulting in an increased plasmon coupling and thus the observed blue shift in the reflected color [46,47,48,49,50].

## 4. Conclusions

In summary, we report tunable hierarchical Ag nanostructures on a stretchable substrate that could potentially be applied to various biomedical sensing applications through micro-actuated movement. Different thicknesses of Ag (20 to 60 nm) were deposited onto a flexible membrane and dewetted utilizing a low-temperature process to create nanoislands. The diameter of the dewetted Ag nanoislands determined the initial color presented by the membrane, where a larger diameter moved it closer to the red portion of the visible spectrum. When a lateral tensile force was applied to the PDMS membrane, a blue shift of more than 100 nm in the visible spectrum range was observed. Potential applications of the tunable hierarchical Ag membrane include biomedical sensing applications such as wearable and implantable sensors that can measure a variety of parameters on or inside the human body. The flexible, epidermal, and biocompatible characteristics of the sensor, with adjustable dimensions from micrometers to millimeters in height and millimeters to centimeters in width and length, provide sufficient evidence of its ability to perform in a variety of biomedical sensing applications, such as the monitoring of swelling and visual indication of traction in fracture treatments. Future work includes studying the spectral shift of the reflected light due to distributed (thus non-uniform) stretching of the nanostructure, investigating fabrication methods to seal the Ag nanoislands in the cavities from oxygen to improve stability and shelf life, and quantifying long-term repeatability and durability of the performance of the sensor. We will also optimize the patterns, number of gratings, and membrane thickness to achieve a larger shift in reflectance spectra to further improve the sensor’s sensitivity. Lastly, we will pursue animal tests and experiments to investigate the reliability of the sensor, and develop real-time image/video processing algorithms to achieve accurate correlation between the tensile force/strain and the reflected color of the sensor.

## Data Availability

Not Applicable.
